# COVID-19 Biomarkers at the Crossroad between Patient Stratification and Targeted Therapy: The Role of Validated and Proposed Parameters

**DOI:** 10.3390/ijms24087099

**Published:** 2023-04-12

**Authors:** Manuela Rizzi, Davide D’Onghia, Stelvio Tonello, Rosalba Minisini, Donato Colangelo, Mattia Bellan, Luigi Mario Castello, Francesco Gavelli, Gian Carlo Avanzi, Mario Pirisi, Pier Paolo Sainaghi

**Affiliations:** 1 Department of Health Sciences, Università del Piemonte Orientale, 28100 Novara, Italy; 2 Department of Translational Medicine, Università del Piemonte Orientale, 28100 Novara, Italy

**Keywords:** COVID-19, biomarkers, red cell distribution width (RDW), D-dimer, ferritin, neutrophil-to-lymphocyte ratio (NLR), C-reactive protein (CRP), interleukin 6 (IL6), IFN-inducible protein 10 (IP10), growth arrest-specific gene 6 (Gas6), SARS-CoV-2 viremia, osteopontin (OPN), calcitonin gene-related peptide (CGRP)

## Abstract

Clinical knowledge about SARS-CoV-2 infection mechanisms and COVID-19 pathophysiology have enormously increased during the pandemic. Nevertheless, because of the great heterogeneity of disease manifestations, a precise patient stratification at admission is still difficult, thus rendering a rational allocation of limited medical resources as well as a tailored therapeutic approach challenging. To date, many hematologic biomarkers have been validated to support the early triage of SARS-CoV-2-positive patients and to monitor their disease progression. Among them, some indices have proven to be not only predictive parameters, but also direct or indirect pharmacological targets, thus allowing for a more tailored approach to single-patient symptoms, especially in those with severe progressive disease. While many blood test-derived parameters quickly entered routine clinical practice, other circulating biomarkers have been proposed by several researchers who have investigated their reliability in specific patient cohorts. Despite their usefulness in specific contexts as well as their potential interest as therapeutic targets, such experimental markers have not been implemented in routine clinical practice, mainly due to their higher costs and low availability in general hospital settings. This narrative review will present an overview of the most commonly adopted biomarkers in clinical practice and of the most promising ones emerging from specific population studies. Considering that each of the validated markers reflects a specific aspect of COVID-19 evolution, embedding new highly informative markers into routine clinical testing could help not only in early patient stratification, but also in guiding a timely and tailored method of therapeutic intervention.

## 1. Background

In December 2019, Chinese researchers first described a cluster of pneumonia cases of unknown origin that rapidly became a global health threat. Since the first clinical reports of a new form of interstitial pneumonia, severe acute respiratory syndrome coronavirus 2 (SARS-CoV-2) has been rapidly identified as the etiological agent responsible for the resulting new coronavirus disease (COVID-19). From a biological point of view, SARS-CoV-2 is a positive-sense, single-stranded RNA virus belonging to the beta coronavirus genus, which shows a very high genetic similarity to other pandemic coronaviruses, namely, SARS-CoV-1 and MERS-CoV (Middle East respiratory syndrome coronavirus), responsible for the SARS and MERS outbreaks in 2002 and 2012, respectively [1,2,3]. The viral genome encodes for 4 structural proteins responsible for virus infectivity and replication, and 16 non-structural proteins accounting for virus-specific functions. Among the 4 structural proteins, a pivotal role is played by the spike (S) protein, which is involved in the binding to the host cell receptors (i.e., angiotensin converting enzyme 2, ACE2) and coreceptors (i.e., heparan sulphate proteoglycans). Furthermore, it is worth noticing that mutations associated with the spike protein are of great interest, as they are able to influence viral transmission and vaccine efficacy [4,5,6].

The main route of infection is represented by respiratory droplets, and SARS-CoV-2 shows a high tropism to the airway epithelium based on ACE2 expression, thus accounting for the widely observed respiratory manifestations of the disease. Moreover, ACE2 expression is not limited to the respiratory system: this host receptor has also been identified in different human tissues, such as the gut, kidneys, heart, blood vessels, and nervous system, where it is believed to contribute to the known extrapulmonary manifestations of COVID-19 [1,4,7,8,9]. 

To date, it is known that most COVID-19 patients develop a mild or moderate disease, while others progress to a more severe illness, finally resulting in death [10,11,12,13]. Furthermore, it has been observed that the patients developing the most severe and critical form of the disease usually experience a highly dysregulated inflammatory response, the so-called cytokine storm, which is recognized as one of the main drivers of the COVID-19-related acute respiratory distress syndrome (ARDS) and multiorgan failure [14,15,16].

Since the beginning of the COVID-19 emergency, many studies have identified advanced age and pre-existing comorbidities as important predictors of mortality in these patients [12,17,18,19,20]. To support the early and correct triage of patients, clinicians soon began to look for early diagnostic tools able to provide a precise stratification of SARS-CoV-2-positive patients upon their hospital admission. As patients with different disease severity levels require different clinical management, reliable stratification biomarkers, defined as measurable, accurate, and reproducible indicators of a biological process [21], should ensure a rational allocation of medical resources, such as isolation and home treatment for asymptomatic and mild patients and hospitalization for moderate-to-severe patients, with a timely transfer to an intensive care unit (ICU) for the most critical ones. To fulfill this clinical need, since the beginning of the pandemic, many biomarkers have been proposed for COVID-19 patients’ stratification. Moreover, due to the hyperinflammatory environment associated with the most severe disease manifestations, several of these molecules not only represent markers of ongoing infection, but could also be supportive tools in assisting pharmacological decisions as well as promising therapeutic targets in severely ill patients. 

This narrative review will present an overview of some of the available biomarkers for COVID-19, focusing both on those already used in clinical practice and on those that have been proposed for the stratification of patients in specific cohort studies, but that have not been yet implemented into the clinical routine. A literature search was conducted by screening PubMed, Google Scholar, and Scops repositories up to February 2023.

## 2. Currently Validated Biomarkers in Clinical Practice

Since the beginning of the pandemic, it has been evident that demographical factors alone, such as age and comorbidities, were not sufficient to drive clinical decisions. Due to the worldwide shortage of medical resources and to the overwhelming pressure on national and international health systems, the need for more precise prognostic predictors has become compelling. The first biomarkers to be introduced into clinical practice for COVID-19 patient stratification were certain hematological parameters, which are easily available in every hospital. The first hematological biomarkers (i.e., white blood cells count, thrombocytopenia, etc.) have been rapidly supported by the validation of some derived ratios (i.e., neutrophil-to-lymphocyte ratio—NLR, platelet-to-lymphocyte ratio—PLR, etc.), allowing for a more precise stratification. As the SARS-CoV-2 infection spread around the world, the scientific knowledge of its pathophysiology grew accordingly, allowing for the validation of new biomarkers that quickly entered in the routine blood tests (i.e., interleukin 6—IL6, etc.) [12,17].

**Figure 1:**
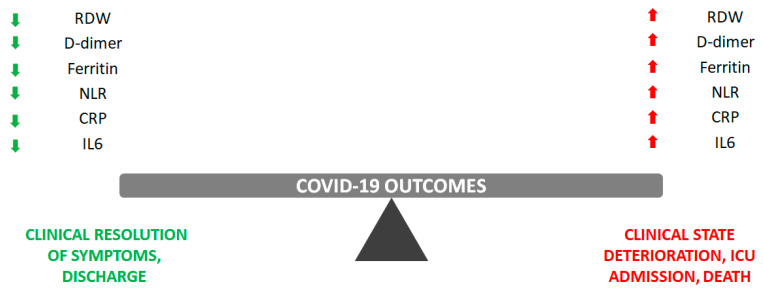
Summary of the most clinically used biomarker (RDW, D-dimer, ferritin, NLR, CRP, and IL6) trajectories in predicting COVID-19 disease evolution.

The following subsections will introduce the most popular biomarkers in clinical practice which have been validated for reliable patient stratification according to expected disease evolution (Figure 1). Some of them, such as C-reactive protein (CRP) and interleukin 6 (IL6), show a direct correlation with the SARS-CoV-2-induced cytokine storm, while the others (red cell distribution width—RDW, D-dimer, ferritin, and neutrophil-to-lymphocyte ratio—NLR) are markers of inflammation, showing high sensitivity but low specificity, as they are known to be elevated in many different pathological conditions.

### 2.1. Red Cell Distribution Width (RDW)

RDW is a low-cost standard component of routine complete blood counts which is automatically generated by many hematological analyzers. It represents the measure of anisocytosis, which represents the heterogeneity of red blood cell size both between different cells and within the same cell during its lifespan [22,23,24,25]. 

Due to its easy availability, this simple hematologic indicator has been investigated as a predictive biomarker in many pathological conditions, such as autoimmune diseases, gastrointestinal disorders, and cancer [26,27,28,29,30,31,32]. Moreover, many existing reports describe RDW as a consistent predictor of all-cause mortality across different study cohorts [22,23,25,33,34]. 

For these reasons, it is not surprising that the prognostic ability of RDW has also been strongly investigated in the context of the COVID-19 pandemic. 

Many research groups have highlighted a strong correlation between elevated RDW and COVID-19 severity, as well as mortality [22,23,24,25,35,36,37]. Furthermore, RDW has been shown to retain its ability to independently predict a negative outcome even after adjustment for the most prevalent confounders, such as age, gender, and other common laboratory parameters and comorbidities [25,33,35,36]. Interestingly, it has been also observed that this hematological parameter displays a complex relationship with underlying COVID-19 pathophysiology: the well known disease-associated hypoxemia, inflammation, and bone marrow overstimulation are all conditions able to induce an increase in RDW, thus supporting its routine evaluation both at admission and for the duration of hospitalization, as well as its adoption as a guiding criterion for early patient stratification. This is due to the fact that those patients with higher RDW at admission or experiencing an RDW increase during their hospital stay are more likely to experience a negative disease evolution resulting in advanced respiratory support, and even ICU admission or death [22,23,36,37].

### 2.2. D-Dimer

D-dimer generally refers to a mixture of peptide fragments with a broad range of molecular weights, deriving from cross-linked fibrin degradation by plasmin. Its plasma half-life is around 8 h, after which it undergoes renal clearance. Due to its proteolytic nature, in physiological conditions, D-dimer is detectable in healthy individuals only in small amounts, slightly increasing with age and pregnancy. On the other hand, when coagulation and fibrinolytic processes are activated following a pathologic insult, its plasma levels increase significantly, accounting for its wide use as an indirect marker of thrombosis [38,39,40,41]. 

For nearly 30 years, this hematologic parameter has been used as a clinical biomarker for patient stratification in several contexts; in fact, it is considered the gold standard for venous thromboembolism prediction in low-to-medium-risk patients, and it is used to guide anticoagulant therapy, to exclude acute aortic dissection, to diagnose and monitor disseminated intravascular coagulation of different origins, and to predict thrombotic complications in septic patients and in those with severe infections [39,40,41,42,43]. Nevertheless, as D-dimer elevation is a common occurrence in different clinical settings, it is noteworthy that it should be considered as a sensitive thrombotic marker with a low specificity whose prediction strength increases when it is used in combination with other biomarkers or diagnostic approaches [41].

As one of the specific features of COVID-19 is represented by the associated vascular disease and thrombosis [38,44,45], it is not surprising that D-dimer, along with fibrinogen, has been investigated as a promising biomarker in this context. 

During the early phases of the pandemic, fibrinogen was investigated as a potential early biomarker able to identify patients at higher risk of developing severe COVID-19. Previous studies have shown that elevated levels of fibrinogen at admission correlate with disease severity, but, in consideration of its dynamic variations during disease evolution (i.e., elevated during the acute phase response vs. reduced upon disseminated coagulopathy), and of its low specificity, no consensus on its clinical use has been reached [46,47]. For these reasons, and considering that D-dimer is the end product of fibrinogen proteolytic degradation, nowadays it is the most widely used biomarker in COVID-19 clinical evaluation.

Autoptic studies on COVID-19 deaths have shown a great prevalence of lung microvascular thrombosis: in light of the observed disease-induced coagulopathy, many studies have described elevated D-dimer levels at admission as an independent predictor of negative disease evolution and in-hospital COVID-19 mortality [38,44,45,48,49,50]. On the other hand, recent studies have highlighted that D-dimer levels below the standard or age-adjusted threshold in SARS-CoV-2 patients referred to the emergency department could be considered as a predictor of a low risk for pulmonary embolism complications [51,52]. 

Even if the involvement of D-dimers in COVID-19 pathophysiology is not fully understood, it has been hypothesized that its increase is a direct consequence of microthrombosis in lung and kidney capillaries, as it should be considered that thromboinflammation is one of the host defense mechanisms commonly activated in response to viral, bacterial, and fungal pathogens [38,45,48,49]. 

Moreover, it should be considered that a high inflammatory milieu is generally associated with marked alterations in blood coagulation tests: severely ill patients, who frequently evolve towards a negative outcome, often experience de-regulated inflammatory responses and hypercytokinemia, thus further supporting the observed rise in D-dimer levels as COVID-19 severity increases [44,45,50,53,54]. Last but not least, it should also be considered that fibrin degradation products are able to induce acute pulmonary dysfunction and display a direct procoagulant effect [38,55]. 

Considering its wide availability through routine hematologic screening, D-dimer evaluation at admission and during the hospital stay could represent a useful tool to monitor disease evolution, allowing for an early identification of patients at greater risk of developing thromboembolic events and a more accurate scheduling of anticoagulant prophylaxis, as it has been demonstrated that the timely administration of anticoagulant drugs in COVID-19 patients with elevated D-dimer levels is directly associated with improved survival [56,57,58,59].

### 2.3. Ferritin

Ferritin is a multisubunit protein characterized by a central nanocage structure, allowing for the storage of iron atoms that can be found in cells and tissues, as well as in serum. Its primary biological function is related to iron storage, but nowadays it is clear that this protein accounts for many other important functions, such as the regulation of iron homeostasis (i.e., it releases iron in case of depletion and binds it in case of excess), protection from invading pathogens (i.e., it reduces iron availability to support bacterial and viral replication) and oxidative stress damage (i.e., it prevents the noxious effects of Haber–Weiss and Fenton reactions) [60,61,62,63,64,65].

Serum ferritin mainly depends on cellular release, and its biological function is essentially related to iron storage, which is why its quantification represents a commonly used approach to support the diagnostic processes of conditions characterized by iron deficiency (i.e., iron-deficiency anemia) or overload (i.e., hereditary hemochromatosis, transfusional iron overload) [60,62,64,65]. Moreover, serum ferritin is recognized as an inflammatory biomarker, and hyperferritinemia is often used to identify high-risk influenza-A-positive patients [64]; subjects with autoimmune conditions, such as adult-onset Still’s disease and systemic lupus erythematosus [60,62,64,65]; and individuals suffering from acute or chronic inflammation, where the increase in serum ferritin results in the so-called anemia of inflammation, a defense mechanism by which the body reduces iron availability to support invading pathogen metabolism [53,60,66].

As the most severe COVID-19 manifestations are frequently associated with a de-regulated inflammatory response, it is not surprising that many research groups have investigated the possible use of ferritin as a reliable biomarker to support the identification of the most high-risk patients.

In the literature, many reports have highlighted a positive relationship between hyperferritinemia and COVID-19 mortality [63,66,67,68,69]. Furthermore, in SARS-CoV-2 patients, elevated serum ferritin has been demonstrated to be able to predict not only in-hospital mortality, but also disease severity and the deterioration of clinical conditions, leading to ICU admission [53,60,61,62,63,66,67,68,69,70].

Despite the clear relationship between hyperferritinemia and COVID-19 disease evolution, the underlying mechanism is still difficult to interpret, with ferritin acting both as a marker and an actor of the inflammatory process. To date, it has been proposed that a vicious loop between ferritin and inflammation exists, with pro-inflammatory cytokines such as IL-6 promoting ferritin release from hepatocytes, Kuppffer cells, and macrophages, while, on the other hand, ferritin itself promotes the expression of different inflammatory mediators [66,67,70].

Considering that ferritin evaluation is now currently included in routine hematologic screening in the emergency department and in the ICU, its use could represent an additional tool for clinicians to stratify SARS-CoV-2-positive patients early, allowing for a more rational resource allocation.

### 2.4. Neutrophil-to-Lymphocyte Ratio (NLR)

NLR is a systemic inflammatory-derived marker representing the ratio of absolute neutrophil count to absolute lymphocyte count. It is known to reflect inflammation progression, which is characterized by an increase in neutrophil count paralleling a decrease in lymphocyte count [71,72,73]. Due to its nature, NLR is a dynamic parameter, possibly reflecting the balance between innate and adaptive immune response, thus allowing for the simultaneous evaluation of both inflammation and pathogen-dependent stress [71,74]. 

As it is a simple and cost-effective marker, it is routinely evaluated in both emergency settings and medical wards to quickly evaluate the clinical status of the patient, thus contributing to risk stratification, especially in case of inflammation-driven or infectious diseases [71,75]. Thanks to its wide availability, NLR is commonly used as a prognostic indicator for many different clinical conditions, such as sepsis, multiorgan failure, pregnancy complications, cardiovascular, liver, and respiratory diseases, and cancer, where a rise in its value is consistent with an increase in severity, leading to a worse prognosis and even death [72,73,75,76,77].

Furthermore, it has been observed that NLR in critically ill patients correlates well with other clinical indices, such as the APACHE (Acute Physiology And Chronic Health Evaluation) II and SOFA (Sequential Organ Failure Assessment) scores [77]; for this reason, it is not surprising that NLR has quickly gained attention as a rapid and cost-effective marker for early patient stratification in the context of the SARS-CoV-2 pandemic. 

Several studies have already highlighted that severely ill COVID-19 patients usually display a sustained increase in NLR at admission as a direct consequence of the hyperinflammatory and immunosuppressive state caused by the viral infection [36,71,73,78,79,80]. Even though there is no consensus about the NLR cut-off to be used in COVID-19 patients’ triage, as its value is known to be influenced by age, ethnicity, and comorbidities [72,74,77], it is noteworthy that it represents an objective parameter for the purpose of identifying those patients needing a close clinical monitoring early and for monitoring their clinical evolution, especially in those situations where the available clinical resources are scarce.

### 2.5. C-Reactive Protein (CRP)

CRP is a liver-produced pattern recognition protein which plays a key role in immunity, being synthesized mainly in response to pro-inflammatory stimuli such as interleukin (IL)-1, IL6, and tumor necrosis factor. 

From a physiological point of view, its role is dual, exerting both pro- and anti-inflammatory actions. In healthy people, CRP circulating levels are nearly undetectable: in case of inflammation, its levels quickly rise, rapidly peaking within 48 h, and, thus, rapidly decrease after inflammation resolution. Such dynamic changes in CRP circulating levels reflect its pathogen-induced liver synthesis, qualifying it as an acute phase reactant whose primary role relies on early complement system activation. It assures host defense while limiting the potentially harmful effects due to the massive activation of the late-stage complement response [20,81,82,83,84,85]. 

Its dynamicity makes CRP a widely used marker in clinics, where it helps to detect acute infections as well as to monitor disease evolution, post-surgical progresses, and treatment responses. Furthermore, there is also evidence that CRP evaluation could be used to evaluate chronic inflammation in vasculitis and rheumatoid arthritis, and that CRP levels slightly higher than normal represent a useful marker for cardiovascular diseases-related inflammation, thus making this protein a very versatile tool in clinical practice, even if its low specificity does not support conclusive diagnoses in the absence of other clinical evidence [81,82,83,85,86]. 

Thanks to its wide availability in routine blood tests performed both in emergency and intensive care settings, as well as medical wards, and to its proven usefulness as a non-specific systemic marker of inflammatory response, CRP has also been investigated in the context of the SARS-CoV-2 pandemic. 

Severe COVID-19 patients usually present with a hyperinflammatory status, and several studies have proposed CRP as a marker of cytokine storm in these patients [20,87]. Furthermore, as it is an acute phase reactant, it has been observed that in SARS-CoV-2 patients, an increase in its circulating levels can be detected in the very early phases of the disease [87,88,89,90], even before lung lesions become detectable by computer tomography [20,91,92]. Thus, it represents a very helpful tool to identify those patients needing immediate attention and closer clinical monitoring. 

Moreover, CRP levels are not only an early stratification marker, but also a valuable tool to predict disease evolution, as higher CRP levels have been detected in COVID-19 progressive patients when compared to stable ones [20,91]. 

Finally, it is noteworthy that CRP levels should be carefully monitored during the entire hospital stay, as it has been observed that its evaluation after 7 days of hospitalization could represent a reliable marker of the treatment response in moderate and severe COVID-19 patients [14,93], highlighting a possible lack of corticosteroid response or, eventually, the development of secondary infections [90,94,95].

Considering the proven usefulness of this non-specific inflammation marker in supporting the early triage of SARS-CoV-2-infected patients, it is worth consideration that its predictive power toward negative COVID-19 evolution is increased when it is combined with other inflammatory markers, such as D-dimer [96], thus supporting its use and implementation in both emergency and general hospital settings.

### 2.6. Interleukin 6 (IL6)

IL6 is a proinflammatory cytokine involved in both innate and immune responses to infection and tissue injuries. Although its main biological function is defensive, an excessive production of such a cytokine leads to the development of different chronic inflammatory diseases, such as rheumatoid arthritis and Castleman’s disease, as well as to the onset of acute hyperinflammation conditions [97,98,99,100]. 

In physiological conditions, circulating IL6 levels are very low, but they undergo a sustained increase in acute conditions, when the cytokine is released by different immune cells and become a classical hallmark of a cytokine storm. Such a rapid increase in IL6 circulating levels has been shown to stimulate the liver to produce and release acute phase proteins, and especially CRP [97,98,100]. The role of IL6 in host defense from pathogen invasion is not limited to the stimulation of acute phase reactant release, but also relies on its ability to activate the coagulation cascade and to orchestrate immune responses. 

When acting as immune regulator, IL6 is able to work on both the innate and acquired arms of the system: on one side, it can de-regulate natural killer and CD8^+^ T cell responses, thus reducing antiviral defenses; on the other side, it can interfere with acquired immune responses by promoting B cell differentiation toward antibody-producing plasma cells and by regulating CD4 T cell differentiation toward Th2 and Th17 lymphocytes [98,100,101,102,103]. 

Considering the complex role of IL6 in immune defenses, and its critical role in hyperinflammation, it is not surprising that this cytokine is also an interesting biomarker and therapeutic target in SARS-CoV-2 infection, a clinical condition that, in its severe form, is generally characterized by a high viral load, hyperinflammation, and poor prognosis. 

Several studies have already highlighted the direct correlation between IL6 levels and COVID-19 severity [14,97,104,105,106]. As IL6 is one of the key regulators of acute phase reactant production, its clinical evaluation has been proven to be useful not only at admission as a predictor of negative outcomes, but also during the entirety of hospitalization, to guide therapeutic interventions. In a recent study, Salton and coworkers demonstrated that IL6 evaluation after 7 days of hospitalization is an independent index of therapeutic response in severely ill patients, as, at that time, it reflects the success of glucocorticoid treatment [14]. 

According to the role of IL6 in the complex COVID-19 pathogenesis, involving not only a deregulated inflammatory and immune response, but also a prothrombotic milieu, this cytokine has also been investigated as a promising therapeutic target. 

Since the beginning of the pandemic, different observational studies and clinical trials have investigated the effectiveness of IL6 signaling inhibitors in preventing ARDS and mortality in SARS-CoV-2-positive patients. In this context, the most studied drug is tocilizumab, a recombinant humanized monoclonal antibody directed to IL6 receptor alpha [101,107,108]. Recent meta-analyses, including the most recently published results of randomized clinical trials performed using a random-effects model to pool the results of the clinically heterogeneous trials, found that tocilizumab administered along with the standard of care therapy was able to reduce both 28-day mortality index and the need of mechanical ventilation and ICU admission, as well as to shorten the time to discharge [97,102,109,110].

## 3. Proposed Biomarkers Not Yet Implemented in Clinical Practice

**Figure 2:**
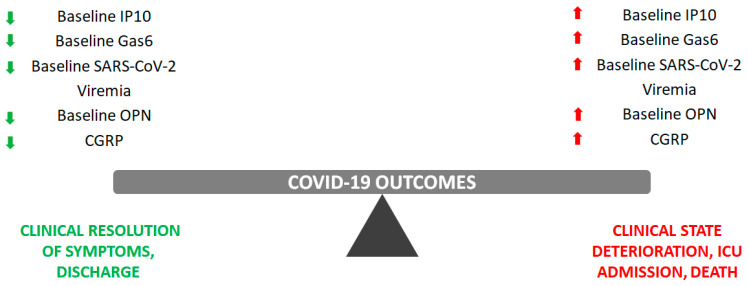
Summary of the most promising biomarker (IP10, Gas6, RNAemia, osteopontin, and CGRP) trajectories in predicting COVID-19 disease evolution.

In addition to the “classical” biomarkers described in the previous section, which are widely used in clinical practice due to their high availability in the context of routine serological examinations, both in emergency departments and medical wards, there are several other biomarkers that have shown good prediction power in specific study populations (Figure 2).

Even if such results originate from heterogeneous clinical cohorts, including patients at different clinical COVID-19 stages and receiving different therapeutic regimens, they are worthy of further investigation to validate them for clinical practice. Once validated, these new biomarkers could be combined with those already being used to define a powerful algorithm that is able to assure the reliable early stratification of SARS-CoV-2-positive patients according to their expected disease evolution, in order to guide clinical decisions toward a more “tailored” approach based on the individual patients’ characteristics.

The following subsections will introduce the most promising biomarkers that have not yet been implemented in clinical practice and which show a good prognostic ability to stratify patients according to their expected disease evolution. Among them, IFN-inducible protein 10 (IP10) is the only one directly related to cytokine storm, while the other protein markers (growth arrest specific protein 6—Gas6, osteopontin—OPN, and calcitonin gene related peptide—CGRP) are aspecific parameters indicating an underlying inflammatory condition.

### 3.1. IFN-Inducible Protein 10 (IP10)

IP10, also known as CXCL10 (C-X-C motif ligand 10), is an interferon (IFN) γ-inducible chemokine that is secreted by several cellular populations of both immune (i.e., T lymphocytes, neutrophils, monocytes) and non-immune (i.e., endothelial cells, fibroblasts, keratinocytes) origin [111,112,113]. As it is secreted in response to cytokine stimulation, high IP10 circulating levels are a well-recognized marker of immune activation, particularly of a Th1-driven immune response to viruses, bacteria, fungi, and other parasites [111,112,113,114]. 

From a biological point of view, this chemokine exerts several functions; the most important ones for immune defense comprise the regulation of leukocyte homing to inflamed tissues and the perpetuation of the inflammatory response, finally resulting in tissue damage and/or cellular apoptosis [111,114]. Interestingly, IP10 has been shown to play a role in several viral infections, showing a pro- (as for human immunodeficiency virus—HIV) or anti- (as for SARS-CoV and Epstein–Barr viruses) infective role depending on the host immune status [111,114]. Furthermore, it has already been investigated in the context of the previous SARS outbreak, during which it was described as a protective immune mediator as its production upon IFN-γ triggering was responsible for the early development of protective T cell responses and virus clearance [111,114,115]. Finally, it is noteworthy that IP10 is a crucial pro-inflammatory mediator in respiratory syndromes, where its expression directly correlates with an adverse prognosis. As a matter of fact, the sustained increase in IP10 production during severe infections was found to induce lymphopenia and T cell response impairment, as well as to exacerbate inflammation, finally resulting in tissue damage and organ dysfunction [111,116,117]. 

Due to the good predictive performance of IP10 in identifying SARS-CoV-positive patients who would undergo a worsening of their clinical conditions [111,116,117], it is not surprising that this chemokine has also been closely investigated in the context of COVID-19, a disease sharing many important pathophysiological features with SARS. 

Today, it is well-recognized that high levels of IP10 during the early phases of COVID-19 are an independent predictor of an adverse clinical outcome, as highlighted by several research groups across the world [93,111,118,119,120,121]. 

Baseline IP10 performs excellently in predicting disease evolution in SARS-CoV-2 positive patients, thus fostering its implementation in clinical practice, where it could be used in combination with more classical biomarkers to improve resource allocation, making it more rational and cost-effective.

### 3.2. Growth Arrest-Specific Gene 6 (Gas6)

Gas6 is a vitamin K-dependent glycoprotein known to be involved in many homeostatic functions, as well as in regulating inflammatory responses. In physiological conditions, plasma Gas6 levels are usually low, while they increase in the case of inflammation [122,123,124,125,126,127,128,129,130]. 

Gas6 biological activities depend on its binding to one of the three members of a family of tyrosine kinase receptors, collectively named TAM (for Tyro-3, Axl, MerTK), which in turn activates different intracellular signaling pathways (i.e., the p38/MAPK, the ERK1/2, the JAK/STAT, and the PI3K/Akt pathways) [131,132,133,134,135,136,137,138]. 

Due to its widely recognized role in immunomodulation, the Gas6/TAM axis has also been investigated in the context of COVID-19, especially considering that one of the distinctive hallmarks of the severe disease is represented by an hyperinflammatory response, accountable for both disease severity and long-term sequelae [3,14,131,139,140,141,142]. 

Since the beginning of the pandemic, many research groups have highlighted a direct correlation between Gas6 plasma levels and COVID-19 severity [143,144,145,146]. Notably, it has been observed that the basal level of Gas6 is that which allows for the best stratification of patients (i.e., those with high Gas6 at admission were most likely to develop the most severe disease) [143,144,145,146]; this observation thus supports the assumption that Gas6 behaves as an acute-phase reactant [128,147] involved in the development of the hyperinflammatory and prothrombotic environment which is usually observed in the most critical patients [148,149]. 

Such experimental evidence supports the reliability of basal Gas6 levels in the early stratification of COVID-19 patients according to their expected negative evolution; on the other hand, studies regarding the predictive ability of the Gas6/TAM axis toward long-term sequelae are still warranted [131], and could represent an interesting starting point to implement currently available disease evolution prediction models. Finally, it should be noted that according to recent in vitro and in vivo evidence highlighting the possible role of Axl in SARS-CoV-2 infection, different studies are ongoing with the aim to repurpose Axl inhibitors as potential anti-COVID-19 drugs [131,150,151], thus supporting the interest in the implementation of Gas6/TAM screening in clinical practice.

### 3.3. SARS-CoV-2 Viremia

The term viremia is used to describe a viral genome that is directly detected in the bloodstream, thus being able to access all body tissues. The assessment of blood viral load has been a useful approach to evaluate the degree of infection, as well as the effectiveness of antiviral treatment, in several viral infections [152,153,154,155,156]. 

Considering the fact that, even if COVID-19 is mainly a pulmonary disease, many extrapulmonary manifestations have been described, both as atypical onset symptoms and as a result of SARS-CoV-2-dependent tissue damage [157,158,159], it is not surprising that viremia detection has also gained interest in this context. 

From a pathophysiological point of view, it could be speculated that the SARS-CoV-2 genomic material that is detected in blood derives from both damage to primary infected tissues (lung or other extrapulmonary tissues, such as the gastrointestinal tract, kidney, heart, and vascular district) and subsequent spread of the virus from these highly infected districts to the systemic circulation, or from active viral replication into the vascular district [160,161]. 

To date, many studies have highlighted an inverse correlation between viremia and humoral immune responses [162,163], as well as a direct association between the presence of SARS-CoV-2 genetic material in blood and COVID-19 severity, with severely ill patients showing a detectable blood viral load associated with both inflammatory markers and clinical indicators of a negative disease trajectory [157,164,165,166,167]. Furthermore, it is known that COVID-19 can manifest with a wide range of symptoms of different severities, so it is possible for some patients to develop an hyperinflammatory response even in the absence of a detectable viremia [167]. 

According to the available literature, it is well accepted that patients experiencing the most severe disease manifestations, with worse prognoses, longer times to recovery, and higher risks of death or ICU admission, have detectable blood SARS-CoV-2 viral loads [157,161,164,168,169,170]. It is noteworthy that, in severe patients, viremia is generally associated with a significant rise in other biomarkers with prognostic significance, such as IL6, CRP, or troponin [54,157,167,169,170], thus supporting the notion that systemic viral spread is not the only driver of the observed adverse outcomes, especially considering the different observed time courses in these events [157,167]. 

In light of the complex nature of COVID-19, viremia evaluation represents a promising biomarker to be implemented in clinical practice, as its integration with other easily achievable hematologic biomarkers and clinical indicators could offer a more precise overview of patients’ disease evolution.

### 3.4. Osteopontin (OPN)

OPN is a small integrin-binding ligand N-link glycoprotein existing both as extracellular matrix protein and as secreted cytokine. It is known to display multiple biological activities, being involved in many physiological processes, such as bone remodeling and immune modulation, as well as pathological conditions, such as cancer, diabetes, nephrolithiasis, and lung and cardiovascular diseases [171,172,173,174,175]. 

In physiological conditions, circulating OPN levels are low, while they undergo a sustained increase during inflammation or cell-mediated immune response activation. In this context, OPN modulates leukocyte differentiation, migration, and activation, leading to cytokine production and release [171,172,173,176,177,178]. Ongoing bacterial and viral infections trigger OPN release and the subsequent Th1 responses, finally resulting in a vicious cycle which perpetuates inflammation. For these reasons, it has already been used as a non-specific marker to monitor the progression of various diseases and to predict a negative outcome in specific conditions, such as sepsis and aneurysmal subarachnoid hemorrhage [171,178,179,180,181,182]. 

Considering OPN’s implications in both inflammatory and immune-mediated responses, it is not surprising that this cytokine has been evaluated as predictive biomarker for COVID-19 severity monitoring. 

To date, it is well established that SARS-CoV-2-induced pneumonia depends on the impairment of monocytes’ metabolism and functions [183,184], and, according to such evidence, several studies have highlighted a direct relationship between increased OPN levels and the severe clinical evolution of SARS-CoV-2-infected patients [171,174,176,179,185]. Moreover, OPN is also correlated with lung fibrotic evolution [186,187,188]; Karabulut Uzunçakmak and coworkers demonstrated that OPN levels are directly associated not only with COVID-19 severity, but also with the development of pulmonary fibrosis, a condition usually observed in the most critical patients [179]. This evidence supports the implementation of OPN evaluation during triage procedures in SARS-CoV-2-positive patients.

### 3.5. Calcitonin Gene-Related Peptide (CGRP)

CGRP is a neuropeptide, existing in two distinct isoforms, whose biological activities appears to be largely overlapping. It is widely expressed in both central and peripheral nervous system, as well as in some non-nervous tissues, such as esophageal Langerhans cells, lymphocytes, and epithelial and endothelial cells [189,190,191,192]. 

From a biological point of view, CGRP is not only a potent vasodilator, but it is also involved in immune regulation. In particular, its synthesis rapidly increases following inflammation, when it is produced by sensory nerves and activated immune cells [189,192]. 

CGRP is known to modulate immune responses by displaying a dual role: on the one hand, it is involved in sustaining inflammation by augmenting cytokine-induced IL6 production [189,192,193], while on the other hand, it has been described as a negative regulator of inflammatory processes, acting by promoting the accumulation and arrest of T cells and antigen-presenting cells, as well as by inhibiting the migration of mature dendritic cells, through the activation of some key signaling mediators, such as PKA, PLC-β1, and PKC [190]. 

Due to its complex role in immune response regulation and its recently highlighted role in bronchial protection [194,195], this vasoactive neuropeptide has also recently gained attention for application in COVID-19 patients. 

Unfortunately, so far, only a few studies have focused on this topic, with conflicting results [194,196,197]. While the most recent one [196] reported a direct correlation between circulating CGRP levels and disease severity, the previous ones [194,197] obtained different results: in particular, one found low serum CGRP levels in COVID-19 patients along with a high RAMP1 (receptor activity-modifying protein 1) lung expression [194], while the other [197] failed to find a direct correlation between this peptide and headache in moderate COVID-19 patients. The observed heterogeneity of the results of these studies can mainly be explained by the different compositions of clinical cohorts, as well as by the different therapeutic regimens adopted. 

The available evidence regarding CGRP’s role as a predictive biomarker for COVID-19 evolution is still scarce, which at present, prevents us from drawing any conclusion, but fosters new studies on the topic. As reported by Rizzi and coworkers [196], this peptide appears to be promising not only for patient stratification at admission, but also for early detection of those patients who have already experienced and/or are experiencing pulmonary and vascular events. This study was monocentric and limited only to non-ICU hospitalized COVID-19 patients with moderate or severe symptoms, thus precluding the generalizability of the obtained conclusions without dedicated studies. Nevertheless, our results support the importance of a tailored therapeutic approach based on a single patient’s specific disease signature retrieved from an analysis of highly informative biomarker panels.

## 4. Conclusions

At the time of writing, we are entering the third year of the COVID-19 pandemic, and this disease still represents a world health concern. Even if mass vaccination campaigns have reduced the mortality rate associated with the SARS-CoV-2 infection, the lack of resolutive therapeutic options makes the need for reliable biomarkers able to predict disease evolution undeniable for the optimization of clinical resource allocation. 

To date, it is well-accepted that COVID-19 can present in many different ways, with a subgroup of patients developing only a very mild disease while others develop a critical illness requiring intensive care and eventually leading to death. In this context, there is a necessity to identify highly accurate and objective parameters to be used to drive patient management during the entire disease course, assuring them timely and effective clinical support.

Furthermore, it should be considered that many of these biomarkers, and especially those most strictly related to cytokine storm, could also be valuable tools to monitor therapeutic responses (i.e., CRP and IL6 after 7 days of hospitalization) and promising direct (i.e., IL6) or indirect (i.e., D-dimer) pharmacological targets in selected patients. Lastly, it is noteworthy that the continuous biotechnological progresses in the field of COVID-19 biomarkers discovery have also led to new and promising findings in terms of possible therapeutic approaches, as demonstrated by the anti-inflammatory and anti-viral activity of heparin [198,199,200], by the ability of IL6 and other IL- and cytokine-signaling inhibitors to improve the disease course [101,102,109,110,201,202,203,204,205] and by the promising antiviral effects of the already existing Gas6/TAM axis inhibitors [131,150,151]. Moreover, several studies are currently focused on both drug repurposing and new drug development, thus representing new potential options to directly or indirectly target several key mediators of COVID-19 pathogenesis [203,206,207,208]. 

Lastly, it should be considered that several recent studies have highlighted that a large proportion of COVID-19 survivors still experience a variety of clinical sequelae for months after the resolution of the acute condition, developing a new clinical condition termed “long COVID” [209,210]. To date, the knowledge about the pathophysiology of long COVID is limited, and its clinical management suffers from a lack of specific diagnostic markers and therapeutic targets. Nevertheless, some recently published studies have started to investigate the potential of different circulating biomarkers in predicting the development of such long-term sequelae, with the aim to develop new therapeutic interventions which are able to ameliorate or even solve the most invalidating symptoms [209,211,212]. As long COVID syndrome is emerging worldwide as an important health concern, the discovery of reliable biomarkers and therapeutic targets deserves further dedicated investigations.

Due to the complex physiopathology of COVID-19, it is undeniable that a single biomarker reflecting all the most striking aspects of the disease does not exist. 

Considering that blood tests are routinely performed at admission and during the entire hospital stay, circulating biomarkers represent an ideal solution to assist in patient triage. As each one of the already validated markers reflects a specific aspect of COVID-19 evolution, embedding new, highly informative markers into routine clinical testing could help in early-risk stratification and to promptly initiate the most appropriate therapeutic intervention.

Furthermore, it should be considered that a correct early stratification of SARS-CoV-2-positive patients at admission is not only mandatory to assure a rational allocation of limited medical resources, but is also a crucial step to assure positive results with immunotherapeutic treatments. In fact, according to the available literature, immunotherapy in COVID-19 is beneficial only in selected patients, while being ineffective or even contraindicated in others. For this reason, the identification of clear numerical cut-offs for reliable biomarkers reflecting the complexity and heterogeneity of the disease would be helpful in recognizing the actual disease stage of progression and in identifying the most relevant pathogenic actors at that stage, thus guiding clinical decisions in terms of targeted pharmacological interventions, which need to be administered at the correct time to the correct patient in order to prevent lethal consequences.

To reach such crucial objectives, it is essential to reduce the economic burden of newly identified biomarkers, allowing for the development of rapid and high-throughput tests and, finally, resulting in the generation of an objective and user-friendly decisional algorithm for the prediction of expected clinical outcomes and therapeutic responses in hospitalized COVID-19 patients.

## Data Availability

Not applicable.

## References

[1] Hernandez A., Costa R.A., Esquer Garrigos Z., Marcelin J.R., Vijayvargiya P. (2022). COVID-19 pathogenesis and clinical manifestations. Infect. Dis. Clin. N. Am..

[2] Lamers M.M., Haagmans B.L. (2022). SARS-CoV-2 pathogenesis. Nat. Rev. Microbiol..

[3] Shafqat A., Shafqat S., Al Salameh S., Kashir J., Alkattan K., Yaqinuddin A. (2022). Mechanistic insights into the immune pathophysiology of COVID-19; an in-depth review. Front. Immunol..

[4] Jamison D.A., Narayanan S.A., Trovão N.S., Guarnieri J.W., Topper M.J., Moraes-Vieira P.M., Zaksas V., Singh K.K., Wurtele E.S., Beheshti A. (2022). A comprehensive SARS-CoV-2 and COVID-19 review, Part 1: Intracellular overdrive for SARS-CoV-2 infection. Eur. J. Hum. Genet..

[5] Attaway A.H., Scheraga R.G., Bhimraj A., Biehl M., Hatipoğlu U. (2021). Severe covid-19 pneumonia: Pathogenesis and clinical management. BMJ.

[6] Hu B., Guo H., Zhou P., Shi Z.-L. (2021). Characteristics of SARS-CoV-2 and COVID-19. Nat. Rev. Microbiol..

[7] Aldè M., Barozzi S., Di Berardino F., Zuccotti G., Consonni D., Ambrosetti U., Socci M., Bertoli S., Battezzati A., Foppiani A. (2022). Prevalence of symptoms in 1512 COVID-19 patients: Have dizziness and vertigo been underestimated thus far?. Intern. Emerg. Med..

[8] Zhang Y., Chen X., Jia L., Zhang Y. (2022). Potential mechanism of SARS-CoV-2-associated central and peripheral nervous system impairment. Acta. Neurol. Scand..

[9] Murgolo N., Therien A.G., Howell B., Klein D., Koeplinger K., Lieberman L.A., Adam G.C., Flynn J., McKenna P., Swaminathan G. (2021). SARS-CoV-2 tropism, entry, replication, and propagation: Considerations for drug discovery and development. PLoS Pathog..

[10] de Morais Batista F., Puga M.A.M., da Silva P.V., Oliveira R., Dos Santos P.C.P., da Silva B.O., Tatara M.B., Tsuha D.H., Dos Santos Pires M.A., Gonçalves C.C.M. (2022). Serum biomarkers associated with SARS-CoV-2 severity. Sci. Rep..

[11] Geraili Z., Hajian-Tilaki K., Bayani M., Hosseini S.R., Khafri S., Ebrahimpour S., Javanian M., Babazadeh A., Shokri M. (2022). Prognostic accuracy of inflammatory markers in predicting risk of ICU admission for COVID-19: Application of time-dependent receiver operating characteristic curves. J. Int. Med. Res..

[12] Shi C., Wang L., Ye J., Gu Z., Wang S., Xia J., Xie Y., Li Q., Xu R., Lin N. (2021). Predictors of mortality in patients with coronavirus disease 2019: A systematic review and meta-analysis. BMC Infect. Dis..

[13] Shang W., Dong J., Ren Y., Tian M., Li W., Hu J., Li Y. (2020). The value of clinical parameters in predicting the severity of COVID-19. J. Med. Virol..

[14] Salton F., Confalonieri P., Campisciano G., Cifaldi R., Rizzardi C., Generali D., Pozzan R., Tavano S., Bozzi C., Lapadula G. (2022). Cytokine profiles as potential prognostic and therapeutic markers in SARS-CoV-2-induced ARDS. J. Clin. Med..

[15] Zanza C., Romenskaya T., Manetti A.C., Franceschi F., La Russa R., Bertozzi G., Maiese A., Savioli G., Volonnino G., Longhitano Y. (2022). Cytokine storm in COVID-19: Immunopathogenesis and therapy. Medicina.

[16] Shcherbak S.G., Anisenkova A.Y., Mosenko S.V., Glotov O.S., Chernov A.N., Apalko S.V., Urazov S.P., Garbuzov E.Y., Khobotnikov D.N., Klitsenko O.A. (2021). Basic predictive risk factors for cytokine storms in COVID-19 patients. Front. Immunol..

[17] Asaduzzaman M.D., Romel Bhuia M., Nazmul Alam Z., Zabed Jillul Bari M., Ferdousi T. (2022). Significance of hemogram-derived ratios for predicting in-hospital mortality in COVID-19: A multicenter study. Health Sci. Rep..

[18] Corradini E., Ventura P., Ageno W., Cogliati C.B., Muiesan M.L., Girelli D., Pirisi M., Gasbarrini A., Angeli P., Querini P.R. (2021). SIMI-COVID-19 Collaborators. Clinical factors associated with death in 3044 COVID-19 patients managed in internal medicine wards in Italy: Results from the SIMI-COVID-19 study of the Italian Society of Internal Medicine (SIMI). Intern. Emerg. Med..

[19] Dessie Z.G., Zewotir T. (2021). Mortality-related risk factors of COVID-19: A systematic review and meta-analysis of 42 studies and 423,117 patients. BMC Infect. Dis..

[20] Li Y., Li H., Song C., Lu R., Zhao Y., Lin F., Han D., Chen L., Pan P., Dai M. (2021). Early prediction of disease progression in patients with severe COVID-19 using C-reactive protein to albumin ratio. Dis. Markers.

[21] Barichello T., Generoso J.S., Singer M., Dal-Pizzol F. (2022). Biomarkers for sepsis: More than just fever and leukocytosis-a narrative review. Crit. Care.

[22] Lucijanić M., Jordan A., Jurin I., Piskač Živković N., Sorić E., Hadžibegović I., Atić A., Stojić J., Rudan D., Jakšić O. (2022). Red cell distribution width is a potent prognostic parameter for in-hospital and post-discharge mortality in hospitalized coronavirus disease 2019 patients: A registry-based cohort study on 3941 patients. Croat. Med. J..

[23] Jandaghian S., Vaezi A., Manteghinejad A., Nasirian M., Vaseghi G., Haghjooy Javanmard S. (2021). Red blood cell distribution width (RDW) as a predictor of in-hospital mortality in COVID-19 patients; a cross sectional study. Arch. Acad. Emerg. Med..

[24] Lippi G., Henry B.M., Sanchis-Gomar F. (2021). Red blood cell distribution is a significant predictor of severe illness in coronavirus disease 2019. Acta. Haematol..

[25] Foy B.H., Carlson J.C.T., Reinertsen E., Padros I., Valls R., Pallares Lopez R., Palanques-Tost E., Mow C., Westover M.B., Aguirre A.D. (2020). Association of red blood cell distribution width with mortality risk in hospitalized adults with SARS-CoV-2 infection. JAMA Netw. Open.

[26] Zinellu A., Mangoni A.A. (2022). Platelet and red blood cell volume indices in patients with rheumatoid arthritis: A systematic review and meta-analysis. Diagnostics.

[27] Bellan M., Soddu D., Zecca E., Croce A., Bonometti R., Pedrazzoli R., Sola D., Rigamonti C., Castello L.M., Avanzi G.C. (2020). Association between red cell distribution width and response to methotrexate in rheumatoid arthritis. Reumatismo.

[28] Liu C., Yang J., Lu Z. (2020). Study on the red blood cell distribution width in connective tissue disease associated with interstitial lung disease. Biomed. Res. Int..

[29] Bellan M., Giubertoni A., Piccinino C., Dimagli A., Grimoldi F., Sguazzotti M., Burlone M.E., Smirne C., Sola D., Marino P. (2019). Red cell distribution width and platelet count as biomarkers of pulmonary arterial hypertension in patients with connective tissue disorders. Dis. Markers.

[30] Haybar H., Pezeshki S.M.S., Saki N. (2019). Evaluation of complete blood count parameters in cardiovascular diseases: An early indicator of prognosis?. Exp. Mol Pathol..

[31] Ai L., Mu S., Hu Y. (2018). Prognostic role of RDW in hematological malignancies: A systematic review and meta-analysis. Cancer Cell Int..

[32] Goyal H., Lippi G., Gjymishka A., John B., Chhabra R., May E. (2017). Prognostic significance of red blood cell distribution width in gastrointestinal disorders. World J. Gastroenterol..

[33] Kaufmann C.C., Ahmed A., Brunner U., Jäger B., Aicher G., Equiluz-Bruck S., Spiel A.O., Funk G.C., Gschwantler M., Fasching P. (2021). Red cell distribution width upon hospital admission predicts short-term mortality in hospitalized patients with COVID-19: A single-center experience. Front. Med..

[34] Lee J.H., Chung H.J., Kim K., Jo Y.H., Rhee J.E., Kim Y.J., Kang K.W. (2013). Red cell distribution width as a prognostic marker in patients with community-acquired pneumonia. Am. J. Emerg. Med..

[35] Banon T., Wortsman J., Ben Moshe S., Gazit S., Peretz A., Ben Tov A., Chodick G., Perez G., Patalon T. (2021). Evaluating red blood cell distribution width from community blood tests as a predictor of hospitalization and mortality in adults with SARS-CoV-2: A cohort study. Ann. Med..

[36] Bellan M., Azzolina D., Hayden E., Gaidano G., Pirisi M., Acquaviva A., Aimaretti G., Aluffi Valletti P., Angilletta R., Arioli R. (2021). Simple parameters from complete blood count predict in-hospital mortality in COVID-19. Dis. Markers.

[37] Lee J.J., Montazerin S.M., Jamil A., Jamil U., Marszalek J., Chuang M.L., Chi G. (2021). Association between red blood cell distribution width and mortality and severity among patients with COVID-19: A systematic review and meta-analysis. J. Med. Virol..

[38] Berger J.S., Kunichoff D., Adhikari S., Ahuja T., Amoroso N., Aphinyanaphongs Y., Cao M., Goldenberg R., Hindenburg A., Horowitz J. (2020). Prevalence and Outcomes of D-Dimer Elevation in Hospitalized Patients With COVID-19. Arterioscler. Thromb. Vasc. Biol..

[39] Johnson E.D., Schell J.C., Rodgers G.M. (2019). The D-dimer assay. Am. J. Hematol..

[40] Favresse J., Lippi G., Roy P.M., Chatelain B., Jacqmin H., Ten Cate H., Mullier F. (2018). D-dimer: Preanalytical, analytical, postanalytical variables, and clinical applications. Crit. Rev. Clin. Lab. Sci..

[41] Weitz J.I., Fredenburgh J.C., Eikelboom J.W. (2017). A test in context: D-dimer. J. Am. Coll. Cardiol..

[42] Li J., Zhou K., Duan H., Yue P., Zheng X., Liu L., Liao H., Wu J., Li J., Hua Y. (2022). Value of D-dimer in predicting various clinical outcomes following community-acquired pneumonia: A network meta-analysis. PLoS ONE.

[43] Soomro A.Y., Guerchicoff A., Nichols D.J., Suleman J., Dangas G.D. (2016). The current role and future prospects of D-dimer biomarker. Eur. Heart J. Cardiovasc. Pharmacother..

[44] He X., Yao F., Chen J., Wang Y., Fang X., Lin X., Long H., Wang Q., Wu Q. (2021). The poor prognosis and influencing factors of high D-dimer levels for COVID-19 patients. Sci. Rep..

[45] Connors J.M., Levy J.H. (2020). COVID-19 and its implications for thrombosis and anticoagulation. Blood.

[46] Sui J., Noubouossie D.F., Gandotra S., Cao L. (2021). Elevated plasma fibrinogen is associated with excessive inflammation and disease severity in COVID-19 patients. Front. Cell Infect. Microbiol..

[47] Thachil J. (2020). The protective rather than prothrombotic fibrinogen in COVID-19 and other inflammatory states. J. Thromb. Haemost..

[48] Chowdary P. (2022). COVID-19 coagulopathy-what should we treat?. Exp. Physiol..

[49] Chocron R., Duceau B., Gendron N., Ezzouhairi N., Khider L., Trimaille A., Goudot G., Weizman O., Alsac J.M., Pommier T. (2021). Critical COVID-19 France investigators. D-dimer at hospital admission for COVID-19 are associated with in-hospital mortality, independent of venous thromboembolism: Insights from a French multicenter cohort study. Arc. Cardiovasc. Dis..

[50] Poudel A., Poudel Y., Adhikari A., Aryal B.B., Dangol D., Bajracharya T., Maharjan A., Gautam R. (2021). D-dimer as a biomarker for assessment of COVID-19 prognosis: D-dimer levels on admission and its role in predicting disease outcome in hospitalized patients with COVID-19. PLoS ONE.

[51] Lin K., Xu K., Daoust R., Taylor J., Rosychuk R.J., Hau J.P., Davis P., Clark G., McRae A.D., Hohl C.M. (2023). Canadian COVID-19 Emergency Department Rapid Response Network (CCEDRRN) investigators for the Network of Canadian Emergency Researchers, the Canadian Critical Care Trials Group. Prognostic association between d-dimer thresholds and 30-day pulmonary embolism diagnosis among emergency department patients with suspected SARS-CoV-2 infection: A Canadian COVID-19 Emergency Department Rapid Response Network study. CJEM.

[52] Revel M.P., Beeker N., Porcher R., Jilet L., Fournier L., Rance B., Chassagnon G., Fontenay M., Sanchez O. (2022). AP-HP /Universities/Inserm COVID-19 research collaboration, AP-HP Covid CDR Initiative. What level of D-dimers can safely exclude pulmonary embolism in COVID-19 patients presenting to the emergency department?. Eur. Radiol..

[53] Qeadan F., Tingey B., Gu L.Y., Packard A.H., Erdei E., Saeed A.I. (2021). Prognostic values of serum ferritin and D-dimer trajectory in patients with COVID-19. Viruses.

[54] Gupta A., Madhavan M.V., Sehgal K., Nair N., Mahajan S., Sehrawat T.S., Bikdeli B., Ahluwalia N., Ausiello J.C., Wan E.Y. (2020). Extrapulmonary manifestations of COVID-19. Nat. Med..

[55] Manwaring D., Curreri P.W. (1980). The role of platelet aggregation and release in fragment D-induced pulmonary dysfunction. Ann. Surg..

[56] Di Castelnuovo A., Costanzo S., Antinori A., Berselli N., Blandi L., Bonaccio M., Cauda R., Guaraldi G., Menicanti L., Mennuni M. (2021). Heparin in COVID-19 patients is associated with reduced in-hospital mortality: The multicenter italian CORIST study. Thromb. Haemost..

[57] Spyropoulos A.C., Goldin M., Giannis D., Diab W., Wang J., Khanijo S., Mignatti A., Gianos E., Cohen M., Sharifova G. (2021). HEP-COVID Investigators. Efficacy and safety of therapeutic-dose heparin vs standard prophylactic or intermediate-dose heparins for thromboprophylaxis in high-risk hospitalized patients with COVID-19: The HEP-COVID randomized clinical trial. JAMA Intern. Med..

[58] Ayerbe L., Risco C., Ayis S. (2020). The association between treatment with heparin and survival in patients with Covid-19. J. Thromb. Thrombolysis.

[59] Tang N., Bai H., Chen X., Gong J., Li D., Sun Z. (2020). Anticoagulant treatment is associated with decreased mortality in severe coronavirus disease 2019 patients with coagulopathy. J. Thromb. Haemost..

[60] Aslan E.S., Aydın H., Tekin Y.K., Keleş S., White K.N., Hekim N. (2023). Association between iron metabolism and SARS-COV-2 infection, determined by ferritin, hephaestin and hypoxia-induced factor-1 alpha levels in COVID-19 patients. Mol. Biol. Rep..

[61] Kaushal K., Kaur H., Sarma P., Bhattacharyya A., Sharma D.J., Prajapat M., Pathak M., Kothari A., Kumar S., Rana S. (2022). Serum ferritin as a predictive biomarker in COVID-19. A systematic review, meta-analysis and meta-regression analysis. J. Crit. Care.

[62] Mahroum N., Alghory A., Kiyak Z., Alwani A., Seida R., Alrais M., Shoenfeld Y. (2022). Ferritin-from iron, through inflammation and autoimmunity, to COVID-19. J. Autoimmun..

[63] DePalma R.G., Hayes V.W., O’Leary T.J. (2021). Optimal serum ferritin level range: Iron status measure and inflammatory biomarker. Metallomics.

[64] Plays M., Müller S., Rodriguez R. (2021). Chemistry and biology of ferritin. Metallomics.

[65] Knovich M.A., Storey J.A., Coffman L.G., Torti S.V., Torti F.M. (2009). Ferritin for the clinician. Blood Rev..

[66] Yadav D., Pvsn K.K., Tomo S., Sankanagoudar S., Charan J., Purohit A., Nag V., Bhatia P., Singh K., Dutt N. (2022). Association of iron-related biomarkers with severity and mortality in COVID-19 patients. J. Trace Elem. Med. Biol..

[67] Volfovitch Y., Tsur A.M., Gurevitch M., Novick D., Rabinowitz R., Mandel M., Achiron A., Rubinstein M., Shoenfeld Y., Amital H. (2022). The intercorrelations between blood levels of ferritin, sCD163, and IL-18 in COVID-19 patients and their association to prognosis. Immunol. Res..

[68] Zhou S., Li H., Li S. (2022). The associations of iron related biomarkers with risk, clinical severity and mortality in SARS-CoV-2 patients: A meta-analysis. Nutrients.

[69] Alroomi M., Rajan R., Omar A.A., Alsaber A., Pan J., Fatemi M., Zhanna K.D., Aboelhassan W., Almutairi F., Alotaibi N. (2021). Ferritin level: A predictor of severity and mortality in hospitalized COVID-19 patients. Immun. Inflamm. Dis..

[70] Cheng L., Li H., Li L., Liu C., Yan S., Chen H., Li Y. (2020). Ferritin in the coronavirus disease 2019 (COVID-19): A systematic review and meta-analysis. J. Clin. Lab. Anal..

[71] Asghar M.S., Akram M., Yasmin F., Najeeb H., Naeem U., Gaddam M., Jafri M.S., Tahir M.J., Yasin I., Mahmood H. (2022). Comparative analysis of neutrophil to lymphocyte ratio and derived neutrophil to lymphocyte ratio with respect to outcomes of in-hospital coronavirus disease 2019 patients: A retrospective study. Front. Med..

[72] Sarkar S., Khanna P., Singh A.K. (2022). The impact of neutrophil-lymphocyte count ratio in COVID-19: A systematic review and meta-analysis. J. Intensive Care Med..

[73] Prozan L., Shusterman E., Ablin J., Mitelpunkt A., Weiss-Meilik A., Adler A., Choshen G., Kehat O. (2021). Prognostic value of neutrophil-to-lymphocyte ratio in COVID-19 compared with Influenza and respiratory syncytial virus infection. Sci. Rep..

[74] Zahorec R. (2021). Neutrophil-to-lymphocyte ratio, past, present and future perspectives. Bratisl. Lek. Listy.

[75] Forget P., Khalifa C., Defour J.P., Latinne D., Van Pel M.C., De Kock M. (2017). What is the normal value of the neutrophil-to-lymphocyte ratio?. BMC Res. Notes.

[76] Ortega-Rojas S., Salazar-Talla L., Romero-Cerdán A., Soto-Becerra P., Díaz-Vélez C., Urrunaga-Pastor D., Maguiña J.L. (2022). The neutrophil-to-lymphocyte ratio and the platelet-to-lymphocyte ratio as predictors of mortality in older adults hospitalized with COVID-19 in Peru. Dis. Markers.

[77] Parthasarathi A., Padukudru S., Arunachal S., Basavaraj C.K., Krishna M.T., Ganguly K., Upadhyay S., Anand M.P. (2022). The role of neutrophil-to-lymphocyte ratio in risk stratification and prognostication of COVID-19: A systematic review and meta-analysis. Vaccines.

[78] Al-Mazedi M.S., Rajan R., Al-Jarallah M., Dashti R., Al Saber A., Pan J., Zhanna K.D., Abdelnaby H., Aboelhassan W., Almutairi F. (2022). Neutrophil to lymphocyte ratio and in-hospital mortality among patients with SARS-CoV-2: A retrospective study. Ann. Med. Surg..

[79] Zeng Z.Y., Feng S.D., Chen G.P., Wu J.N. (2021). Predictive value of the neutrophil to lymphocyte ratio for disease deterioration and serious adverse outcomes in patients with COVID-19: A prospective cohort study. BMC Infect. Dis..

[80] Li X., Liu C., Mao Z., Xiao M., Wang L., Qi S., Zhou F. (2020). Predictive values of neutrophil-to-lymphocyte ratio on disease severity and mortality in COVID-19 patients: A systematic review and meta-analysis. Crit. Care.

[81] Mosquera-Sulbaran J.A., Pedreañez A., Carrero Y., Callejas D. (2021). C-reactive protein as an effector molecule in Covid-19 pathogenesis. Rev. Med. Virol..

[82] Dyer E.M., Waterfield T., Baynes H. (2019). How to use C-reactive protein. Arch. Dis. Child Educ. Pract. Ed..

[83] Marnell L., Mold C., Du Clos T.W. (2005). C-reactive protein: Ligands, receptors and role in inflammation. Clin. Immunol..

[84] Black S., Kushner I., Samols D. (2004). C-reactive protein. J. Biol. Chem..

[85] Clyne B., Olshaker J.S. (1999). The C-reactive protein. J. Emerg. Med..

[86] Agrawal A. (2005). CRP after 2004. Mol. Immunol..

[87] Luo X., Zhou W., Yan X., Guo T., Wang B., Xia H., Ye L., Xiong J., Jiang Z., Liu Y. (2020). Prognostic value of C-reactive protein in patients with coronavirus 2019. Clin. Infect. Dis..

[88] Bouayed M.Z., Laaribi I., Chatar C.E.M., Benaini I., Bouazzaoui M.A., Oujidi Y., Berrichi S., El Aidouni G., Bkiyar H., Abda N. (2022). C-reactive protein (CRP): A poor prognostic biomarker in COVID-19. Front. Immunol..

[89] Stringer D., Braude P., Myint P.K., Evans L., Collins J.T., Verduri A., Quinn T.J., Vilches-Moraga A., Stechman M.J., Pearce L. (2021). The role of C-reactive protein as a prognostic marker in COVID-19. Int. J. Epidemiol..

[90] Wang L. (2020). C-reactive protein levels in the early stage of COVID-19. Med. Mal. Infect..

[91] Zhang J.N., Gao Y., Wang X.T., Li N.N., Du X., Tang Y.J., Lai Q.Q., Chen P.F., Yue C.S., Wu J.H. (2022). Lymphocyte-C-reactive protein ratio can differentiate disease severity of COVID-19 patients and serve as an assistant screening tool for hospital and ICU admission. Front. Immunol..

[92] Tan C., Huang Y., Shi F., Tan K., Ma Q., Chen Y., Jiang X., Li X. (2020). C-reactive protein correlates with computed tomographic findings and predicts severe COVID-19 early. J. Med. Virol..

[93] Rizzi M., Costanzo M., Tonello S., Matino E., Casciaro F.G., Croce A., Rizzi E., Zecca E., Pedrinelli A., Vassia V. (2022). Prognostic markers in hospitalized COVID-19 patients: The role of IP-10 and C-reactive protein. Dis. Markers.

[94] Luan Y.Y., Yin C.H., Yao Y.M. (2021). Update. Advances on C-reactive protein in COVID-19 and other viral infections. Front. Immunol..

[95] Chen W., Zheng K.I., Liu S., Yan Z., Xu C., Qiao Z. (2020). Plasma CRP level is positively associated with the severity of COVID-19. Ann. Clin. Microbiol. Antimicrob..

[96] Smilowitz N.R., Kunichoff D., Garshick M., Shah B., Pillinger M., Hochman J.S., Berger J.S. (2021). C-reactive protein and clinical outcomes in patients with COVID-19. Eur. Heart J..

[97] Kang S., Kishimoto T. (2021). Interplay between interleukin-6 signaling and the vascular endothelium in cytokine storms. Exp. Mol. Med..

[98] Narazaki M., Kishimoto T. (2018). The two-faced cytokine IL-6 in host defense and diseases. Int. J. Mol. Sci..

[99] Kishimoto T. (2010). IL-6: From its discovery to clinical applications. Int. Immunol..

[100] Rachman A., Rinaldi I. (2006). Coagulopathy in dengue infection and the role of interleukin-6. Acta Med. Indones..

[101] Bryushkova E.A., Skatova V.D., Mutovina Z.Y., Zagrebneva A.I., Fomina D.S., Kruglova T.S., Akopyan A.A., Strazhesko I.D., Lukyanov S.A., Tkacheva O.N. (2022). Tocilizumab, netakimab, and baricitinib in patients with mild-to-moderate COVID-19: An observational study. PLoS ONE.

[102] Zizzo G., Tamburello A., Castelnovo L., Laria A., Mumoli N., Faggioli P.M., Stefani I., Mazzone A. (2022). Immunotherapy of COVID-19: Inside and beyond IL-6 signalling. Front. Immunol..

[103] Scheller J., Chalaris A., Schmidt-Arras D., Rose-John S. (2011). The pro- and anti-inflammatory properties of the cytokine interleukin-6. Biochim. Biophys. Acta.

[104] Sayah W., Berkane I., Guermache I., Sabri M., Lakhal F.Z., Yasmine Rahali S., Djidjeli A., Lamara Mahammed L., Merah F., Belaid B. (2021). Interleukin-6, procalcitonin and neutrophil-to-lymphocyte ratio: Potential immune-inflammatory parameters to identify severe and fatal forms of COVID-19. Cytokine.

[105] Udomsinprasert W., Jittikoon J., Sangroongruangsri S., Chaikledkaew U. (2021). Circulating levels of interleukin-6 and interleukin-10, but not tumor necrosis factor-alpha, as potential biomarkers of severity and mortality for COVID-19: Systematic review with meta-analysis. J. Clin. Immunol..

[106] Mojtabavi H., Saghazadeh A., Rezaei N. (2020). Interleukin-6 and severe COVID-19: A systematic review and meta-analysis. Eur. Cytokine Netw..

[107] Recovery Collaborative Group (2021). Tocilizumab in patients admitted to hospital with COVID-19 (RECOVERY): A randomised, controlled, open-label, platform trial. Lancet.

[108] Rosas I.O., Bräu N., Waters M., Go R.C., Hunter B.D., Bhagani S., Skiest D., Aziz M.S., Cooper N., Douglas I.S. (2021). Tocilizumab in hospitalized patients with severe Covid-19 pneumonia. N. Engl. J. Med..

[109] Wang Y., Zhu K., Dai R., Li R., Li M., Lv X., Yu Q. (2022). Specific Interleukin-1 inhibitors, specific interleukin-6 inhibitors, and GM-CSF blockades for COVID-19 (at the edge of sepsis): A systematic review. Front. Pharmacol..

[110] Zhang J., Chen C., Yang Y., Yang J. (2022). Effectiveness of tocilizumab in the treatment of hospitalized adults COVID-19: A systematic review and meta-analysis. Medicine.

[111] Elemam N.M., Talaat I.M., Maghazachi A.A. (2022). CXCL10 chemokine: A critical player in RNA and DNA viral infections. Viruses.

[112] Antonelli A., Ferrari S.M., Giuggioli D., Ferrannini E., Ferri C., Fallahi P. (2014). Chemokine (C-X-C motif) ligand (CXCL)10 in autoimmune diseases. Autoimmun. Rev..

[113] Lee E.Y., Lee Z.H., Song Y.W. (2013). The interaction between CXCL10 and cytokines in chronic inflammatory arthritis. Autoimmun. Rev..

[114] Liu M., Guo S., Hibbert J.M., Jain V., Singh N., Wilson N.O., Stiles J.K. (2011). CXCL10/IP-10 in infectious diseases pathogenesis and potential therapeutic implications. Cytokine Growth Factor. Rev..

[115] Chen J., Subbarao K. (2007). The Immunobiology of SARS. Annu. Rev. Immunol..

[116] Jiang Y., Xu J., Zhou C., Wu Z., Zhong S., Liu J., Luo W., Chen T., Qin Q., Deng P. (2005). Characterization of cytokine/chemokine profiles of severe acute respiratory syndrome. Am. J. Respir. Crit. Care Med..

[117] Tang N.L., Chan P.K., Wong C.K., To K.F., Wu A.K., Sung Y.M., Hui D.S., Sung J.J., Lam C.W. (2005). Early enhanced expression of interferon-inducible protein-10 (CXCL-10) and other chemokines predicts adverse outcome in severe acute respiratory syndrome. Clin. Chem..

[118] Basheer M., Saad E., Kananeh M., Asad L., Khayat O., Badarne A., Abdo Z., Arraf N., Milhem F., Bassal T. (2022). Cytokine patterns in COVID-19 patients: Which cytokines predict mortality and which protect against?. Curr. Issues Mol. Biol..

[119] Tripathy A.S., Vishwakarma S., Trimbake D., Gurav Y.K., Potdar V.A., Mokashi N.D., Patsute S.D., Kaushal H., Choudhary M.L., Tilekar B.N. (2021). Pro-inflammatory CXCL-10, TNF-α, IL-1β, and IL-6: Biomarkers of SARS-CoV-2 infection. Arch. Virol..

[120] Chen Y., Wang J., Liu C., Su L., Zhang D., Fan J., Yang Y., Xiao M., Xie J., Xu Y. (2020). IP-10 and MCP-1 as biomarkers associated with disease severity of COVID-19. Mol. Med..

[121] Yang Y., Shen C., Li J., Yuan J., Wei J., Huang F., Wang F., Li G., Li Y., Xing L. (2020). Plasma IP-10 and MCP-3 levels are highly associated with disease severity and predict the progression of COVID-19. J. Allergy. Clin. Immunol..

[122] Bellan M., Cittone M.G., Tonello S., Rigamonti C., Castello L.M., Gavelli F., Pirisi M., Sainaghi P.P. (2019). Gas6/TAM system: A key modulator of the interplay between inflammation and fibrosis. Int. J. Mol. Sci..

[123] Law L.A., Graham D.K., Di Paola J., Branchford B.R. (2018). GAS6/TAM pathway signaling in hemostasis and thrombosis. Front. Med..

[124] Sainaghi P.P., Bellan M., Lombino F., Alciato F., Carecchio M., Galimberti D., Fenoglio C., Scarpini E., Cantello R., Pirisi M. (2017). Growth arrest specific 6 concentration is increased in the cerebrospinal fluid of patients with Alzheimer’s disease. J. Alzheimer’s Dis..

[125] van der Meer J.H., van der Poll T., van’t Veer C. (2014). TAM receptors, Gas6, and protein S: Roles in inflammation and hemostasis. Blood.

[126] Sainaghi P.P., Collimedaglia L., Alciato F., Molinari R., Sola D., Ranza E., Naldi P., Monaco F., Leone M., Pirisi M. (2013). Growth arrest specific gene 6 protein concentration in cerebrospinal fluid correlates with relapse severity in multiple sclerosis. Mediators Inflamm..

[127] Alciato F., Sainaghi P.P., Sola D., Castello L., Avanzi G.C. (2010). TNF-alpha, IL-6, and IL-1 expression is inhibited by GAS6 in monocytes/macrophages. J. Leukoc. Biol..

[128] Hurtado B., de Frutos P.G. (2010). GAS6 in systemic inflammatory diseases: With and without infection. Crit. Care.

[129] Alciato F., Sainaghi P.P., Castello L., Bergamasco L., Carnieletto S., Avanzi G.C. (2008). Development and validation of an ELISA method for detection of growth arrest specific 6 (GAS6) protein in human plasma. J. Immunoass. Immunochem..

[130] Sainaghi P.P., Collimedaglia L., Alciato F., Leone M.A., Puta E., Naldi P., Castello L., Monaco F., Avanzi G.C. (2008). Elevation of Gas6 protein concentration in cerebrospinal fluid of patients with chronic inflammatory demyelinating polyneuropathy (CIDP). J. Neurol. Sci..

[131] Rizzi M., Tonello S., D’Onghia D., Sainaghi P.P. (2023). Gas6/TAM axis involvement in modulating inflammation and fibrosis in COVID-19 patients. Int. J. Mol. Sci..

[132] Poświata A., Kozik K., Miączyńska M., Zdżalik-Bielecka D. (2022). Endocytic trafficking of GAS6-AXL complexes is associated with sustained AKT activation. Cell. Mol. Life Sci..

[133] Bellan M., Dimagli A., Piccinino C., Giubertoni A., Ianniello A., Grimoldi F., Sguazzotti M., Nerviani A., Barini M., Carriero A. (2020). Role of Gas6 and TAM receptors in the identification of cardiopulmonary involvement in systemic sclerosis and scleroderma spectrum disorders. Dis. Markers.

[134] Bellan M., Quaglia M., Nerviani A., Mauro D., Lewis M., Goegan F., Gibbin A., Pagani S., Salmi L., Molinari L. (2021). Increased plasma levels of Gas6 and its soluble tyrosine kinase receptors Mer and Axl are associated with immunological activity and severity of lupus nephritis. Clin. Exp. Rheumatol..

[135] Li M., Ye J., Zhao G., Hong G., Hu X., Cao K., Wu Y., Lu Z. (2019). Gas6 attenuates lipopolysaccharide-induced TNF-α expression and apoptosis in H9C2 cells through NF-κB and MAPK inhibition via the Axl/PI3K/Akt pathway. Int. J. Mol. Med..

[136] Bellan M., Pirisi M., Sainaghi P.P. (2016). The Gas6/TAM System and Multiple Sclerosis. Int. J. Mol. Sci..

[137] Lemke G. (2013). Biology of the TAM receptors. Cold Spring Harb. Perspect. Biol..

[138] Fernández-Fernández L., Bellido-Martín L., García de Frutos P. (2008). Growth arrest-specific gene 6 (GAS6). An outline of its role in haemostasis and inflammation. Thromb. Haemost..

[139] Baricich A., Borg M.B., Cuneo D., Cadario E., Azzolina D., Balbo P.E., Bellan M., Zeppegno P., Pirisi M., Cisari C. (2021). Midterm functional sequelae and implications in rehabilitation after COVID-19: A cross-sectional study. Eur. J. Phys. Rehabil. Med..

[140] Bellan M., Baricich A., Patrucco F., Zeppegno P., Gramaglia C., Balbo P.E., Carriero A., Amico C.S., Avanzi G.C., Barini M. (2021). Long-term sequelae are highly prevalent one year after hospitalization for severe COVID-19. Sci. Rep..

[141] Gustine J.N., Jones D. (2021). Immunopathology of hyperinflammation in COVID-19. Am. J. Pathol..

[142] Leisman D.E., Ronner L., Pinotti R., Taylor M.D., Sinha P., Calfee C.S., Hirayama A.V., Mastroiani F., Turtle C.J., Harhay M.O. (2020). Cytokine elevation in severe and critical COVID-19: A rapid systematic review, meta-analysis, and comparison with other inflammatory syndromes. Lancet Respir. Med..

[143] Tonello S., Rizzi M., Matino E., Costanzo M., Casciaro G.F., Croce A., Rizzi E., Zecca E., Pedrinelli A., Vassia V. (2022). Baseline plasma Gas6 protein elevation predicts adverse outcomes in hospitalized COVID-19 patients. Dis. Markers.

[144] de Bruin S., Bos L.D., van Roon M.A., Tuip-de Boer A.M., Schuurman A.R., Koel-Simmelinck M.J.A., Bogaard H.J., Tuinman P.R., van Agtmael M.A., Hamann J. (2021). Clinical features and prognostic factors in Covid-19: A prospective cohort study. EBioMedicine.

[145] Huckriede J., Anderberg S.B., Morales A., de Vries F., Hultström M., Bergqvist A., Ortiz-Pérez J.T., Sels J.W., Wichapong K., Lipcsey M. (2021). Evolution of NETosis markers and DAMPs have prognostic value in critically ill COVID-19 patients. Sci. Rep..

[146] Morales A., Rojo Rello S., Cristóbal H., Fiz-López A., Arribas E., Marí M., Tutusaus A., de la Cal-Sabater P., Nicolaes G.A.F., Ortiz-Pérez J.T. (2021). Growth arrest-specific factor 6 (GAS6) is increased in COVID-19 patients and predicts clinical outcome. Biomedicines.

[147] Ekman C., Linder A., Akesson P., Dahlbäck B. (2010). Plasma concentrations of Gas6 (growth arrest specific protein 6) and its soluble tyrosine kinase receptor sAxl in sepsis and systemic inflammatory response syndromes. Crit. Care.

[148] Levy J.H., Iba T., Olson L.B., Corey K.M., Ghadimi K., Connors J.M. (2021). COVID-19: Thrombosis, thromboinflammation, and anticoagulation considerations. Int. J. Lab. Hematol..

[149] Hanff T.C., Mohareb A.M., Giri J., Cohen J.B., Chirinos J.A. (2020). Thrombosis in COVID-19. Am. J. Hematol..

[150] Maarifi G., Martin M.F., Zebboudj A., Boulay A., Nouaux P., Fernandez J., Lagisquet J., Garcin D., Gaudin R., Arhel N.J. (2022). Identifying enhancers of innate immune signaling as broad-spectrum antivirals active against emerging viruses. Cell Chem. Biol..

[151] Bohan D., Van Ert H., Ruggio N., Rogers K.J., Badreddine M., Aguilar Briseño J.A., Elliff J.M., Rojas Chavez R.A., Gao B., Stokowy T. (2021). Phosphatidylserine receptors enhance SARS-CoV-2 infection. PLoS Pathog..

[152] Reynolds L., Franco R., Prados M., Rodgers J.B., Hand D.T., Walter L.A. (2022). Hepatitis C active viremia over time in an ED-based testing programme: Impact, disparities and surveillance tool. J. Viral. Hepat..

[153] Tosiano M.A., Jacobs J.L., Shutt K.A., Cyktor J.C., Mellors J.W. (2019). A simpler and more sensitive single-copy HIV-1 RNA assay for quantification of persistent HIV-1 viremia in individuals on suppressive antiretroviral therapy. J. Clin. Microbiol..

[154] Conway J.M., Perelson A.S. (2016). Residual Viremia in Treated HIV+ Individuals. PLoS Comput. Biol.

[155] de la Cruz-Hernández S.I., Flores-Aguilar H., González-Mateos S., López-Martinez I., Alpuche-Aranda C., Ludert J.E., del Angel R.M. (2013). Determination of viremia and concentration of circulating nonstructural protein 1 in patients infected with dengue virus in Mexico. Am. J. Trop. Med. Hyg..

[156] Grant P.R., Garson J.A., Tedder R.S., Chan P.K., Tam J.S., Sung J.J. (2003). Detection of SARS coronavirus in plasma by real-time RT-PCR. N. Engl. J. Med..

[157] Elrobaa I.H., New K.J. (2021). COVID-19: Pulmonary and Extra Pulmonary Manifestations. Front. Public Health.

[158] Finsterer J., Scorza F.A., Scorza C.A., Fiorini A.C. (2021). Extrapulmonary onset manifestations of COVID-19. Clinics.

[159] Yazdanpanah F., Garg A., Shadman S., Asmarz H.Y. (2021). Literature review of COVID-19, pulmonary and extrapulmonary disease. Am. J. Med. Sci..

[160] Ram-Mohan N., Kim D., Zudock E.J., Hashemi M.M., Tjandra K.C., Rogers A.J., Blish C.A., Nadeau K.C., Newberry J.A., Quinn J.V. (2022). Stanford COVID-19 Biobank Study Group. SARS-CoV-2 RNAemia predicts clinical deterioration and extrapulmonary complications from COVID-19. Clin. Infect. Dis..

[161] van Riel D., Embregts C.W.E., Sips G.J., van den Akker J.P.C., Endeman H., van Nood E., Raadsen M., Bauer L., van Kampen J., Molenkamp R. (2021). Temporal kinetics of RNAemia and associated systemic cytokines in hospitalized COVID-19 patients. Msphere.

[162] Gutmann C., Takov K., Burnap S.A., Singh B., Ali H., Theofilatos K., Reed E., Hasman M., Nabeebaccus A., Fish M. (2021). SARS-CoV-2 RNAemia and proteomic trajectories inform prognostication in COVID-19 patients admitted to intensive care. Nat. Commun..

[163] Eberhardt K.A., Meyer-Schwickerath C., Heger E., Knops E., Lehmann C., Rybniker J., Schommers P., Eichenauer D.A., Kurth F., Ramharter M. (2020). RNAemia corresponds to disease severity and antibody response in hospitalized COVID-19 patients. Viruses.

[164] Rizzi M., Patrucco F., Trevisan M., Faolotto G., Mercandino A., Strola C., Ravanini P., Costanzo M., Tonello S., Matino E. (2022). Baseline plasma SARS-CoV-2 RNA detection predicts an adverse COVID-19 evolution in moderate to severe hospitalized patients. Panminerva Med..

[165] Colagrossi L., Antonello M., Renica S., Merli M., Matarazzo E., Travi G., Vecchi M., Colombo J., Muscatello A., Grasselli G. (2021). SARS-CoV-2 RNA in plasma samples of COVID-19 affected individuals: A cross-sectional proof-of-concept study. BMC Infect. Dis.

[166] Miki S., Sasaki H., Horiuchi H., Miyata N., Yoshimura Y., Miyazaki K., Matsumura T., Takahashi Y., Suzuki T., Matano T. (2021). On-admission SARS-CoV-2 RNAemia as a single potent predictive marker of critical condition development and mortality in COVID-19. PLoS ONE.

[167] Fajnzylber J., Regan J., Coxen K., Corry H., Wong C., Rosenthal A., Worrall D., Giguel F., Piechocka-Trocha A., Atyeo C. (2020). SARS-CoV-2 viral load is associated with increased disease severity and mortality. Nat. Commun..

[168] Kawasuji H., Morinaga Y., Tani H., Yoshida Y., Takegoshi Y., Kaneda M., Murai Y., Kimoto K., Ueno A., Miyajima Y. (2022). SARS-CoV-2 RNAemia with a higher nasopharyngeal viral load is strongly associated with disease severity and mortality in patients with COVID-19. J. Med. Virol..

[169] Chen X., Zhao B., Qu Y., Chen Y., Xiong J., Feng Y., Men D., Huang Q., Liu Y., Yang B. (2020). Detectable serum severe acute respiratory syndrome coronavirus 2 viral load (RNAemia) is closely correlated with drastically elevated interleukin 6 level in critically ill patients with coronavirus disease 2019. Clin. Infect. Dis..

[170] Roy-Vallejo E., Cardeñoso L., Triguero-Martínez A., Chicot Llano M., Zurita N., Ávalos E., Barrios A., Hernando J., Ortiz J., Rodríguez-García S.C. (2022). SARS-CoV-2 viremia precedes an IL6 response in severe COVID-19 patients: Results of a longitudinal prospective cohort. Front. Med..

[171] Reisner A., Blackwell L.S., Sayeed I., Myers H.E., Wali B., Heilman S., Figueroa J., Lu A., Hussaini L., Anderson E.J. (2022). Osteopontin as a biomarker for COVID-19 severity and multisystem inflammatory syndrome in children: A pilot study. Exp. Biol. Med..

[172] Bellan M., Piccinino C., Tonello S., Minisini R., Giubertoni A., Sola D., Pedrazzoli R., Gagliardi I., Zecca E., Calzaducca E. (2021). Role of osteopontin as a potential biomarker of pulmonary arterial hypertension in patients with systemic sclerosis and other connective tissue diseases (CTDs). Pharmaceuticals.

[173] Cappellano G., Abreu H., Raineri D., Scotti L., Castello L., Vaschetto R., Chiocchetti A. (2021). High levels of circulating osteopontin in inflammatory lung disease regardless of Sars-CoV-2 infection. EMBO Mol. Med.

[174] Hayek S.S., Roderburg C., Blakely P., Launius C., Eugen-Olsen J., Tacke F., Ktena S., Keitel V., Leudde M., Giamarellos-Bourboulis E.J. (2021). Circulating osteopontin levels and outcomes in patients hospitalized for COVID-19. J. Clin. Med..

[175] Shirakawa K., Sano M. (2021). Osteopontin in cardiovascular diseases. Biomolecules.

[176] Varim C., Demirci T., Cengiz H., Hacibekiroglu I., Tuncer F.B., Cokluk E., Toptan H., Karabay O., Yildirim I. (2021). Relationship between serum osteopontin levels and the severity of COVID-19 infection. Wien. Klin. Wochenschr..

[177] Icer M.A., Gezmen-Karadag M. (2018). The multiple functions and mechanisms of osteopontin. Clin. Biochem..

[178] Shi L., Shi L., Wang X., He J. (2018). Regulatory roles of osteopontin in production of monocyte-origin MCP-1. Cell Transpl..

[179] Karabulut Uzunçakmak S., Aksakal A., Kerget F., Aydın P., Halıcı Z. (2023). Evaluation of IGFBP5 expression and plasma osteopontin level in COVID-19 patients. Adv. Med. Sci..

[180] Nakatsuka Y., Shiba M., Nishikawa H., Terashima M., Kawakita F., Fujimoto M., Suzuki H., PSEED Group (2018). Acute-phase plasma osteopontin as an independent predictor for poor outcome after aneurysmal subarachnoid hemorrhage. Mol. Neurobiol..

[181] Roderburg C., Benz F., Cardenas D.V., Lutz M., Hippe H.J., Luedde T., Trautwein C., Frey N., Koch A., Tacke F. (2015). Persistently elevated osteopontin serum levels predict mortality in critically ill patients. Crit. Care.

[182] Lund S.A., Giachelli C.M., Scatena M. (2009). The role of osteopontin in inflammatory processes. J. Cell Commun. Signal..

[183] Bai G., Furushima D., Niki T., Matsuba T., Maeda Y., Takahashi A., Hattori T., Ashino Y. (2021). High levels of the cleaved form of galectin-9 and osteopontin in the plasma are associated with inflammatory markers that reflect the severity of COVID-19 pneumonia. Int. J. Mol. Sci..

[184] Gibellini L., De Biasi S., Paolini A., Borella R., Boraldi F., Mattioli M., Lo Tartaro D., Fidanza L., Caro-Maldonado A., Meschiari M. (2020). Altered bioenergetics and mitochondrial dysfunction of monocytes in patients with COVID-19 pneumonia. EMBO Mol. Med..

[185] Tonello S., D’Onghia D., Apostolo D., Matino E., Costanzo M., Casciaro G.F., Croce A., Rizzi E., Zecca E., Pedrinelli A.R. (2023). Baseline plasma osteopontin protein elevation predicts adverse outcomes in hospitalized COVID-19 patients. Viruses.

[186] Hatipoglu O.F., Uctepe E., Opoku G., Wake H., Ikemura K., Ohtsuki T., Inagaki J., Gunduz M., Gunduz E., Watanabe S. (2021). Osteopontin silencing attenuates bleomycin-induced murine pulmonary fibrosis by regulating epithelial-mesenchymal transition. Biomed. Pharmacother..

[187] Pardo A., Gibson K., Cisneros J., Richards T.J., Yang Y., Becerril C., Yousem S., Herrera I., Ruiz V., Selman M. (2005). Up-regulation and profibrotic role of osteopontin in human idiopathic pulmonary fibrosis. PLoS Med..

[188] Takahashi F., Takahashi K., Okazaki T., Maeda K., Ienaga H., Maeda M., Kon S., Uede T., Fukuchi Y. (2001). Role of osteopontin in the pathogenesis of bleomycin-induced pulmonary fibrosis. Am. J. Respir. Cell Mol. Biol..

[189] Khodabakhsh P., Asgari Taei A., Mohseni M., Bahrami Zanjanbar D., Khalili H., Masoumi K., Haji Abbas Shirazi A., Dargahi L. (2021). Vasoactive peptides: Role in COVID-19 pathogenesis and potential use as biomarkers and therapeutic targets. Arch. Med. Res..

[190] Holzmann B. (2013). Modulation of immune responses by the neuropeptide CGRP. Amino Acids.

[191] Luo D., Zhang Y.W., Peng W.J., Peng J., Chen Q.Q., Li D., Deng H.W., Li Y.J. (2008). Transient receptor potential vanilloid 1-mediated expression and secretion of endothelial cell-derived calcitonin gene-related peptide. Regul. Pept..

[192] Sakuta H., Inaba K., Muramatsu S. (1995). Calcitonin gene-related peptide enhances cytokine-induced IL-6 production by fibroblasts. Cell. Immunol..

[193] Robertson C.E. (2020). Could CGRP Antagonists Be Helpful in the Fight Against COVID-19?. Headache.

[194] Ochoa-Callejero L., García-Sanmartín J., Villoslada-Blanco P., Íñiguez M., Pérez-Matute P., Pujadas E., Fowkes M.E., Brody R., Oteo J.A., Martínez A. (2021). Circulating levels of calcitonin gene-related peptide are lower in COVID-19 patients. J. Endocr. Soc..

[195] Cottrell G.S., Padilla B., Pikios S., Roosterman D., Steinhoff M., Grady E.F., Bunnett N.W. (2007). Post-endocytic sorting of calcitonin receptor-like receptor and receptor activity-modifying protein 1. J. Biol. Chem..

[196] Rizzi M., Tonello S., Morani F., Rizzi E., Casciaro G.F., Matino E., Costanzo M., Zecca E., Croce A., Pedrinelli A. (2022). CGRP plasma levels correlate with the clinical evolution and prognosis of hospitalized acute COVID-19 patients. Viruses.

[197] Bolay H., Karadas Ö., Oztürk B., Sonkaya R., Tasdelen B., Bulut T.D.S., Gülbahar Ö., Özge A., Baykan B. (2021). HMGB1, NLRP3, IL-6 and ACE2 levels are elevated in COVID-19 with headache: A window to the infection-related headache mechanism. J. Headache Pain..

[198] Cardillo G., Viggiano G.V., Russo V., Mangiacapra S., Cavalli A., Castaldo G., Agrusta F., Bellizzi A., Amitrano M., Iannuzzo M. (2021). Antithrombotic and anti-inflammatory effects of fondaparinux and enoxaparin in hospitalized COVID-19 patients: The FONDENOXAVID Study. J. Blood Med..

[199] Buijsers B., Yanginlar C., Maciej-Hulme M.L., de Mast Q., van der Vlag J. (2020). Beneficial non-anticoagulant mechanisms underlying heparin treatment of COVID-19 patients. EBioMedicine.

[200] Gozzo L., Viale P., Longo L., Vitale D.C., Drago F. (2020). The potential role of heparin in patients with COVID-19: Beyond the anticoagulant effect. A Review. Front. Pharmacol..

[201] Albuquerque A.M., Eckert I., Tramujas L., Butler-Laporte G., McDonald E.G., Brophy J.M., Lee T.C. (2023). Effect of tocilizumab, sarilumab, and baricitinib on mortality among patients hospitalized for COVID-19 treated with corticosteroids: A systematic review and meta-analysis. Clin. Microbiol. Infect..

[202] Barkas F., Christaki E., Liberopoulos E., Kosmidou M., Milionis H. (2022). Anakinra in COVID-19: A step closer to the cure. Eur. J. Intern. Med..

[203] Ceramella J., Iacopetta D., Sinicropi M.S., Andreu I., Mariconda A., Saturnino C., Giuzio F., Longo P., Aquaro S., Catalano A. (2022). Drugs for COVID-19: An Update. Molecules.

[204] Cherian J.J., Eerike M., Bagepally B.S., Das S., Panda S. (2022). Efficacy and safety of baricitinib and tocilizumab in hospitalized patients with COVID-19: A comparison using systematic review and meta-analysis. Front. Pharmacol..

[205] Kyriazopoulou E., Huet T., Cavalli G., Gori A., Kyprianou M., Pickkers P., Eugen-Olsen J., Clerici M., Veas F., Chatellier G. (2021). Effect of anakinra on mortality in patients with COVID-19: A systematic review and patient-level meta-analysis. Lancet Rheumatol..

[206] Singh D.D., Han I., Choi E.H., Yadav D.K. (2023). A Clinical Update on SARS-CoV-2: Pathology and Development of Potential Inhibitors. Curr. Issues Mol. Biol..

[207] Peng H., Ding C., Jiang L., Tang W., Liu Y., Zhao L., Yi Z., Ren H., Li C., He Y. (2022). Discovery of potential anti-SARS-CoV-2 drugs based on large-scale screening in vitro and effect evaluation in vivo. Sci. China Life. Sci..

[208] Dittmar M., Lee J.S., Whig K., Segrist E., Li M., Kamalia B., Castellana L., Ayyanathan K., Cardenas-Diaz F.L., Morrisey E.E. (2021). Drug repurposing screens reveal cell-type-specific entry pathways and FDA-approved drugs active against SARS-Cov-2. Cell Rep..

[209] Lai Y.J., Liu S.H., Manachevakul S., Lee T.A., Kuo C.T., Bello D. (2023). Biomarkers in long COVID-19: A systematic review. Front. Med..

[210] Batiha G.E., Al-Kuraishy H.M., Al-Gareeb A.I., Welson N.N. (2022). Pathophysiology of Post-COVID syndromes: A new perspective. Virol. J..

[211] Queiroz M.A.F., Neves P.F.M.D., Lima S.S., Lopes J.D.C., Torres M.K.D.S., Vallinoto I.M.V.C., Bichara C.D.A., Dos Santos E.F., de Brito M.T.F.M., da Silva A.L.S. (2022). Cytokine profiles associated with acute COVID-19 and long COVID-19 syndrome. Front. Cell. Infect. Microbiol..

[212] Patel M.A., Knauer M.J., Nicholson M., Daley M., Van Nynatten L.R., Martin C., Patterson E.K., Cepinskas G., Seney S.L., Dobretzberger V. (2022). Elevated vascular transformation blood biomarkers in long-COVID indicate angiogenesis as a key pathophysiological mechanism. Mol. Med..

